# *CEMIP* as a potential biomarker and therapeutic target for breast cancer patients

**DOI:** 10.7150/ijms.58067

**Published:** 2022-02-07

**Authors:** Jinqi Xue, Xudong Zhu, Xinbo Qiao, Yulun Wang, Jiawen Bu, Xiaoying Zhang, Qingtian Ma, Lu Liang, Lisha Sun, Caigang Liu

**Affiliations:** Department of Oncology, Shengjing Hospital of China Medical University, Shenyang, Liaoning Province, 110004, China.

**Keywords:** Breast cancer, *CEMIP*, Metastasis, Prognosis, Biomarker

## Abstract

**Purpose:** We aimed to evaluate whether *CEMIP* plays any role in the survival outcome of breast cancer (BC) patients, as well as to explore the regulatory mechanism of CEMIP in BC.

**Methods:** We evaluated the expression and prognostic effect of *CEMIP* in BC patients using the Oncomine, GEPIA, UALCAN, and Kaplan-Meier plotter databases. Additionally, we detected *CEMIP* mRNA and protein levels in BC and normal tissues via PCR and western blotting analyses. Through immunochemistry analysis, we quantified *CEMIP* expression in 233 samples from BC patients. We then analyzed the link between the survival outcomes and CEMIP expression based on these clinical samples. Furthermore, we explored the immune-related molecules regulated by *CEMIP* and its coexpressed genes using the STRING database.

**Results:** CEMIP expression was higher in BC tissues than in normal tissues. Patients with high *CEMIP* mRNA levels had a worse survival outcome. Similarly, patients expressing CEMIP had significantly shorter overall survival and disease-free survival than those not expressing the protein (*P* < 0.01). Some lymphocytes, immune inhibitors, immune stimulators, MHC molecules, chemokines*,* and chemokine receptors can be regulated by* CEMIP*, and *CEMIP* and its coexpressed genes can participate in the hyaluronan biosynthetic process, hyaluronan catabolic process, and other related biological processes in the progression of BC.

**Conclusion:** Compared to normal tissues, BC tissues had higher number of *CEMIP* transcripts. CEMIP expression was associated with an adverse prognosis.* CEMIP* and its coexpressed genes can participate in the progression of BC. Therefore, *CEMIP* may be a potential biomarker for the treatment of BC patients.

## Introduction

Breast cancer (BC) is the most frequently diagnosed cancer worldwide 1. It is estimated that more than 3.8 million women in the United States have a history of invasive BC, with nearly 270000 women being newly diagnosed every year 2. Although given the standardization of systemic chemotherapy as the gold-standard approach for most molecular subtypes along with rapid advances in early detection and comprehensive therapy, the number of BC patients continues to grow despite an increasing number of new diagnoses. However, numerous patients suffer from metastasis, relapse, or drug resistance, causing treatment failure 3. These severe situations urge us to explore more advanced means of early diagnosis and treatment, such as novel therapeutic targets.

Cell migration-inducing and hyaluronan-binding protein (*CEMIP*), also known as *TMEM2L* and *KIAA1199*, is a 150 kDa protein with an N-terminal secretion signal peptide. It was first reported as an inner ear protein, and genetic mutations in *CEMIP* led to nonsyndromic hearing loss 4. *CEMIP* has also been reported to depolymerize hyaluronic acid (HA) 5, and overexpression of* CEMIP* can lead to resistance to cell immortalization and carcinogenesis in normal human cells 6. In recent years, its role in carcinogenesis has been observed in various cancers including gastric cancer 7-8, pancreatic cancer 9-10, hepatocellular carcinoma, colorectal cancer*,* and colon cancer 11-14. Overexpression of* CEMIP* has also been observed in BC 15-17 and ovarian cancer 18.

However, further research on *CEMIP* in BC prognosis has rarely been reported, and the underlying mechanism by which *CEMIP* affects BC progression is still unknown. Herein, we aimed to explore *CEMIP* expression in the survival outcomes of BC patients and to determine the relationships between *CEMIP* expression and other clinicopathological characteristics in BC patients. With the help of public databases, we explored the expression of* CEMIP* in large sample size cohorts and then examined the expression level of *CEMIP* in BC tissues collected from surgery patients. These data were then analyzed to explore the clinical implications of *CEMIP* expression in BC.

## Methods

### Oncomine database

The Oncomine database (http://www.oncomine.org) was used to analyze the expression of *CEMIP* mRNA transcripts in different cancer types 19. Additionally, the expression levels of *CEMIP* mRNA transcripts in BC and normal tissues among different research subgroups were explored. Finally, we preliminarily analyzed the genes coexpressed with *CEMIP* in the Ginestier Breast and Schmidt Breast studies.

### GOBO database

Expression of *CEMIP* in the different subtypes of breast tumor and BC cell lines was further explored using the GOBO database (GOBO; http://co.bmc.lu.se/gobo/gsa.pl).

### UALCAN database

The UALCAN database (ualcan.path.uab.edu) was used to analyze the levels of *CEMIP* mRNA transcripts in BC based on the subgroups of sample types, individual cancer stages, major subclasses [with triple-negative BC (TNBC) types], and the effect of CEMIP expression on BC patient survival 20. In addition, the level of DNA methylation of *CEMIP* in BC tissues was also explored.

### Kaplan-Meier plotter

The Kaplan-Meier plotter (http://kmplot.com) was used to analyze the prognostic effect of high *CEMIP* mRNA expression on overall survival (OS) and post-progression survival (PPS) of BC patients 21,22.

### RNA extraction and qRT-PCR

Trizol (Thermo Fisher Scientific Company) was used to extract total RNA from BC and normal breast specimens. After reverse transcription, *CEMIP* mRNA expression was detected by SYBR Premix Taq II (Takara, RR820A, Japan). The primer sequences for *CEMIP* and *GAPDH* were: *CEMIP* primer sequence: forward, 5'-GGAGAGTTCCAAGCAGCA 3'; reverse, 5'-CGTCAATCACCACCACCT-3'; *GAPDH* primer sequence: forward, 5'-CCTTCCGTGTCCCCACT-3'; reverse, 5'-GCCTGCTTCACCACCTTC-3'.

### Western blotting

MCF7, SKBR3, MDA-MB-231, and MCF10A cells were harvested using trypsinization and washed with PBS twice before lysis. Along with the 20 BC and cancer-adjacent specimens, samples were lysed with NP40 lysis and PMSF. Proteins were then separated on a 12% SDS-PAGE gel and transferred to PVDF membrane, followed by blocking with 5% fat-free dry milk for 1 h. Membranes were incubated in a shaker with rabbit monoclonal anti-human CEMIP antibody (Abcam, ab98947) overnight at 4 °C. After washing, the membranes were probed with HRP-conjugated goat anti-rabbit IgG (Zhong Shan Jin Qiao, China) for 1 h at room temperature. These bands were visualized using an enhanced chemiluminescent reagent (Thermo Fisher Scientific, USA).

### BC cell lines and cell culture

The human BC cell lines MCF7, MCF-10A, SK-BR3, and MDA-MB-231 were purchased from American Type Culture Collection (ATCC, Manassas, VA, USA). These BC cells were separately cultured in 10% fetal bovine serum-containing DMEM as well as McCoy's 5A and L15 media, following the manufacturers' instructions, in an atmosphere containing 5% CO_2_ at 37 °C, according to American Type Culture Collection (ATCC) recommendations.

### Patients and BC tissue specimens

For the clinical study, we evaluated breast tissue samples (TMA sample set) from 233 patients who underwent surgery at the China Medical University Affiliated Hospital. Incidences of histologically confirmed ductal BC were recruited from 2006 to 2008. No patient exhibited distant metastases at the time of surgery. Patients with a history of other solid tumors, radiotherapy, chemotherapy, or neoadjuvant chemotherapy were excluded. Clinical information was obtained through a telephone, electronic record system, or in-person visit in outpatient settings. Fresh tumor tissue samples and adjacent normal tissues from 20 patients with breast carcinoma were obtained and stored in liquid nitrogen for western blotting. All patients signed informed consent forms. All samples were validated by expert pathologists. This protocol was approved by China Medical University Institutional Review Board [2020PS171K(X1)].

### Immunohistochemistry and evaluation

All 233 BC specimens were fixed in 4% formaldehyde, embedded in paraffin, and then sectioned at 4 μm. Sections were rehydrated with a graded ethanol series after deparaffinization with xylene followed by Tris-buffered saline (TBS). Tissues were then incubated at 4 °C overnight with antibody against CEMIP (Novus, NBP2-50336UV). After that, BC sections were incubated with a secondary antibody at 37 °C for approximately 50 min. Immunohistochemical staining was performed using a DAB kit for 10 min.

Total CEMIP expression was classified semiquantitatively according to the following criteria: 0 if <1 % of tumor cells expressed CEMIP, 1+ if expression occurred in ≥1 and <5% of tumor cells, 2+ if ≥5% and <10% of tumor cells expressed *CEMIP*, and 3+ if ≥10% did so. The scores of 2+ and 3+ were considered to indicate *CEMIP* positive expression, whereas scores of 0 and 1+ were considered to indicate *CEMIP* negative expression.

### cBioPortal database

The cBioPortal database (http://www.cbioportal.org) was utilized to analyze the genetic variations of *CEMIP* 23.

### MEXPRESS database

The MEXPRESS database (https://mexpress.be/) was utilized to analyze DNA methylation of *CEMIP* in primary BC tissues and normal tissues.

### TISIDB database

The TISIDB database (http://cis.hku.hk/TISIDB) was utilized to explore the correlations between CEMIP expression and immune-related molecules 24.

### Gene coexpressed analysis

Genes coexpressed with *CEMIP* were constructed from the Coexpedia website (http://www.coexpedia.org/) and STRING database version 10.0. Thereafter, we used the STRING database to perform Gene Ontology (GO) enrichment analyses of all the selected genes.

### Statistical analysis

Age, T grade, N grade, menopausal status, ER, PR, HER2, Ki67 index, and other clinicopathological characteristics among BC patients were analyzed using a Chi-square test. Survival curves were generated using the Kaplan-Meier test with the SPSS23.0 software. Disease-free survival (DFS) was defined as the interval from operation to local recurrence or distant metastasis. OS was defined as the interval from surgery to patient death. All statistical tests were two-sided. Significance was set at *P* <0.05, Analyses were performed using the SPSS software (version 23.0; SPSS Inc., IL, USA). The Kaplan-Meier plotter was used to evaluate how *CEMIP* mRNA expression influenced the survival outcome of BC patients.

## Results

### Analysis of differential expression of *CEMIP* in BC

**Figure 1:**
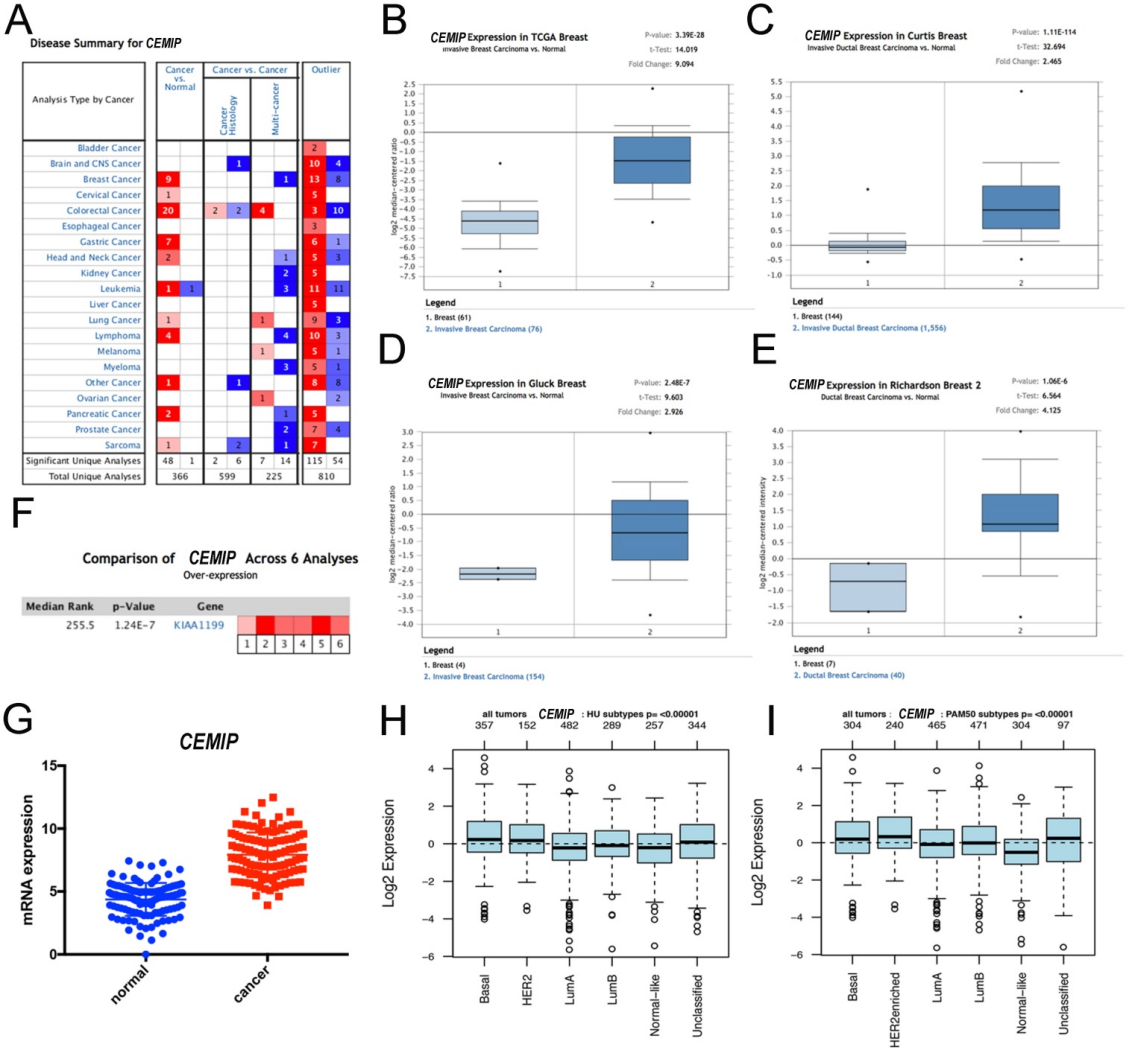
** Exploration the expression of CEMIP in BC tissues and normal breast tissues. A:** The level of *CEMIP* mRNA in different cancer types gained from Oncomine database. The graph presented the number of datasets with statistically significant target genes with increased (red) or reduced expression (blue). **B-E:** Comparison of *CEMIP* mRNA expression in BC tissues and normal tissues in different study subgroups. **F:** Meta-analysis of multiple study datasets for comparison of *CEMIP* expression between BC tissues and normal breast tissues. **G:** The expression of *CEMIP* mRNA in BC tissues and normal breast tissues gained from TCGA database. **H-I:** The level of *CEMIP* mRNA expression in BC patients with different molecular subgroups in GOBO database.

Using Oncomine, we first conducted an overall analysis of the expression of *CEMIP* in multiple cancers and diseases (Figure 1A). We subsequently investigated the expression of *CEMIP* in BC, focusing on the differential expression between cancer and normal tissues. Oncomine analysis revealed that *CEMIP* expression was significantly higher in cancer tissues than in normal samples. Based on the Curtis Breast Statistics dataset, *CEMIP* transcripts were elevated 2.465-fold in BC samples when compared with normal breast tissues (P = 1.11 × 10^-14^) 25 (Figure 1C) and were 2.926-fold elevated in ductal carcinoma *in situ* samples as compared with normal tissues (P = 2.48 × 10^-7^) in Gluck's study 26 (Figure 1D). In a dataset derived from Richardson's study, *CEMIP* levels were elevated 4.125-fold in ductal breast carcinoma compared to normal breast tissues (P = 1.06 × 10^-6^) 2 (Figure 1E). To obtain a more comprehensive conclusion, we conducted a meta-analysis of multiple study datasets including six datasets, TCGA Breast, Cutris Breast, Gluck Breast, Richardson Breast 2, Radvanyi Breast, and Ma Breast 4. This meta-analysis showed a significantly higher expression of *CEMIP* in BC (Figure 1F). Additionally, we explored *CEMIP* expression based on TCGA research network, which also displayed higher (9.094-fold) levels of *CEMIP* in cancer compared to normal breast tissues (Figure 1B,1G). GOBO analyses also validated that *CEMIP* has a significantly high expression in all subtypes of BC (Figure 1H-1I).

**Figure 2:**
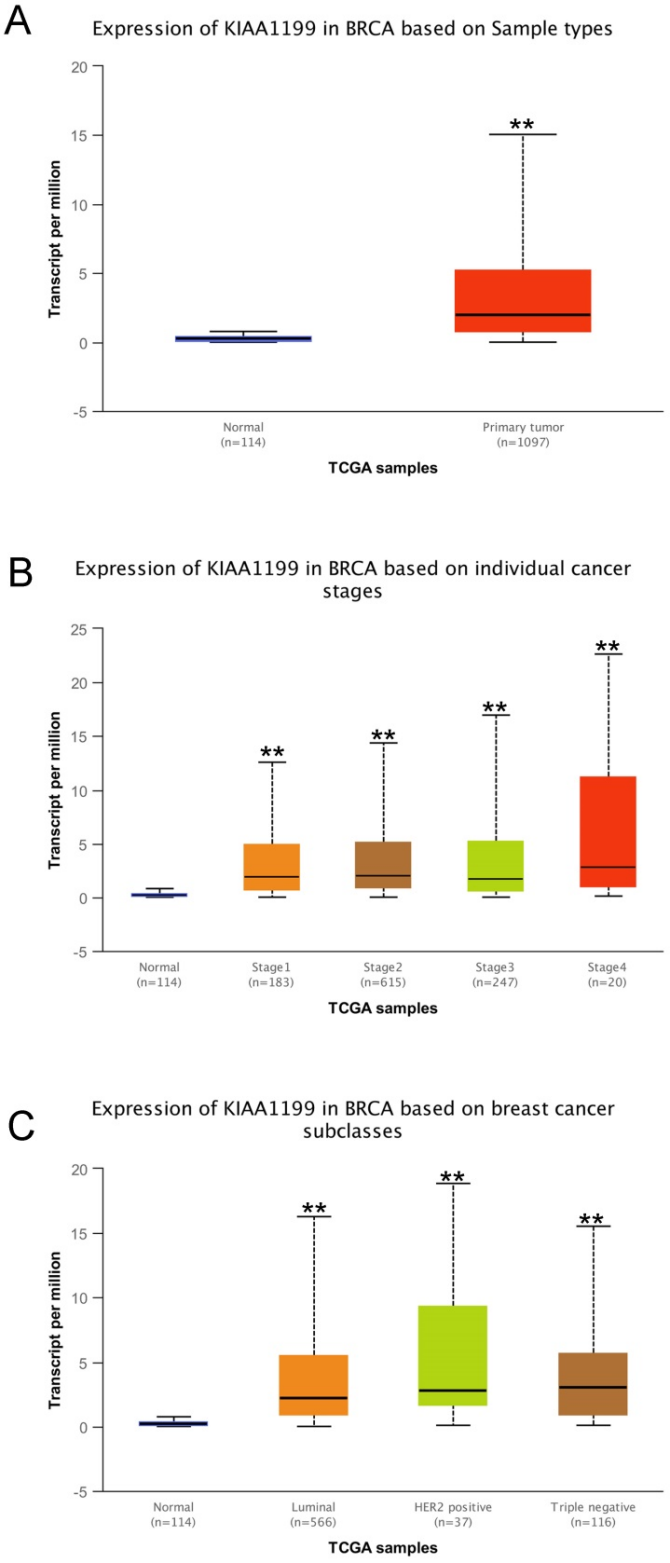
** Analysis of *CEMIP* mRNA expression in BC by UALCAN database.** Expression of *CEMIP* in BC based on different sample types **(A)**, individual cancer stages** (B)**, and BC subclasses **(C)**.

Furthermore, we explored the expression of *CEMIP* in BC using the UALCAN database. *CEMIP* mRNA levels were higher in cancer tissues than in normal tissues (Figure 2A). We also found that *CEMIP* mRNA expression level was significantly higher in breast cancer tissues of different individual stage than in normal tissues (Figure 2B). Out of the four BC subclasses, TNBC patients exhibited the highest *CEMIP* mRNA expression (Figure 2C).

### Exploration of the effect of high expression of *CEMIP* mRNA on the survival outcomes of BC

**Figure 3:**
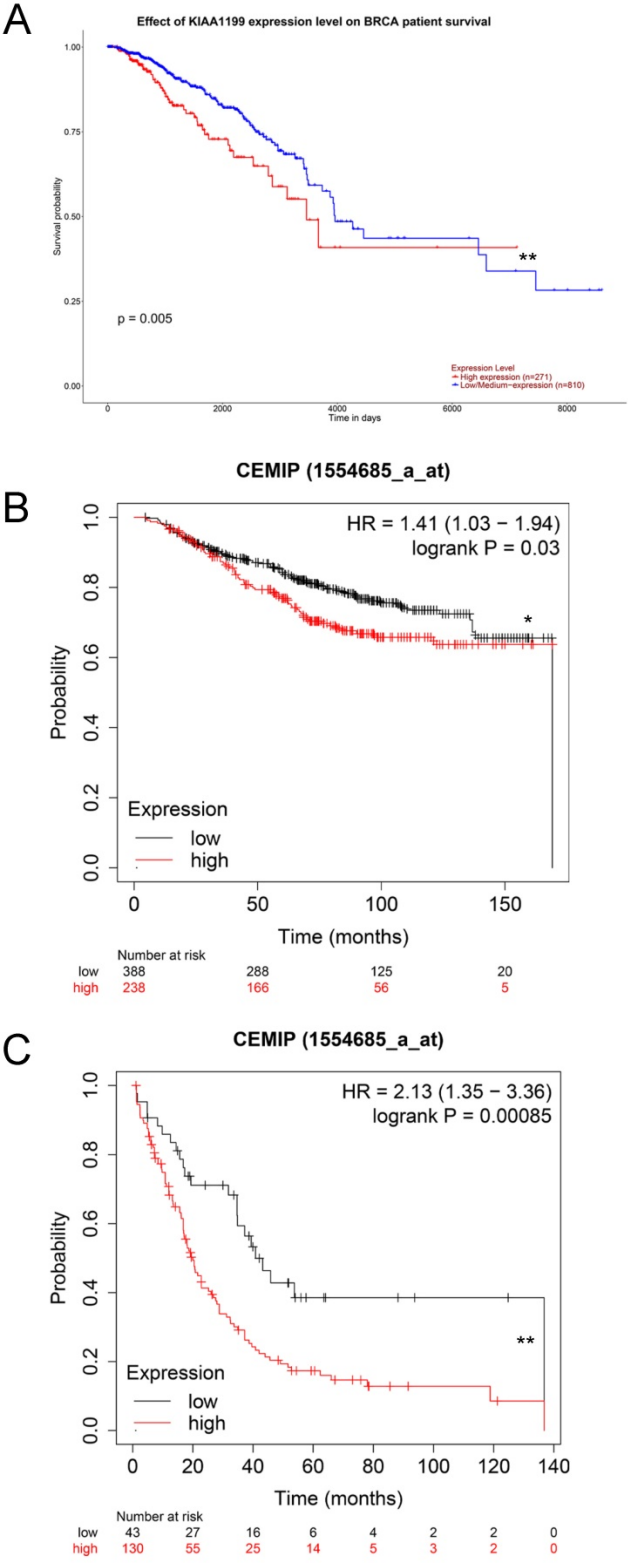
** Effect of CEMIP expression on the prognosis of BC patients. A:** Effect of CEMIP expression in the overall survival of BC patients in UALCAN database. **B:** Effect of CEMIP expression in the overall survival of BC patients in Kaplan-Meier plotter database. **C:** Effect of CEMIP expression in the post-progression survival of BC patients in Kaplan-Meier plotter database.

We used the UALCAN database to explore the effect of high *CEMIP* mRNA expression on the prognosis of BC patients. *CEMIP* mRNA expression was significantly related to a shorter survival time (*P* = 0.005, Figure 3A). Furthermore, we explored the Kaplan-Meier plotter database; *CEMIP* mRNA expression was found to be significantly related to a shorter overall survival (*P* = 0.03, Figure 3B) and post-progression survival (*P* = 0.00085, Figure 3C).

### Expression levels of *CEMIP* is associated with progressive BC malignancy

**Figure 4:**
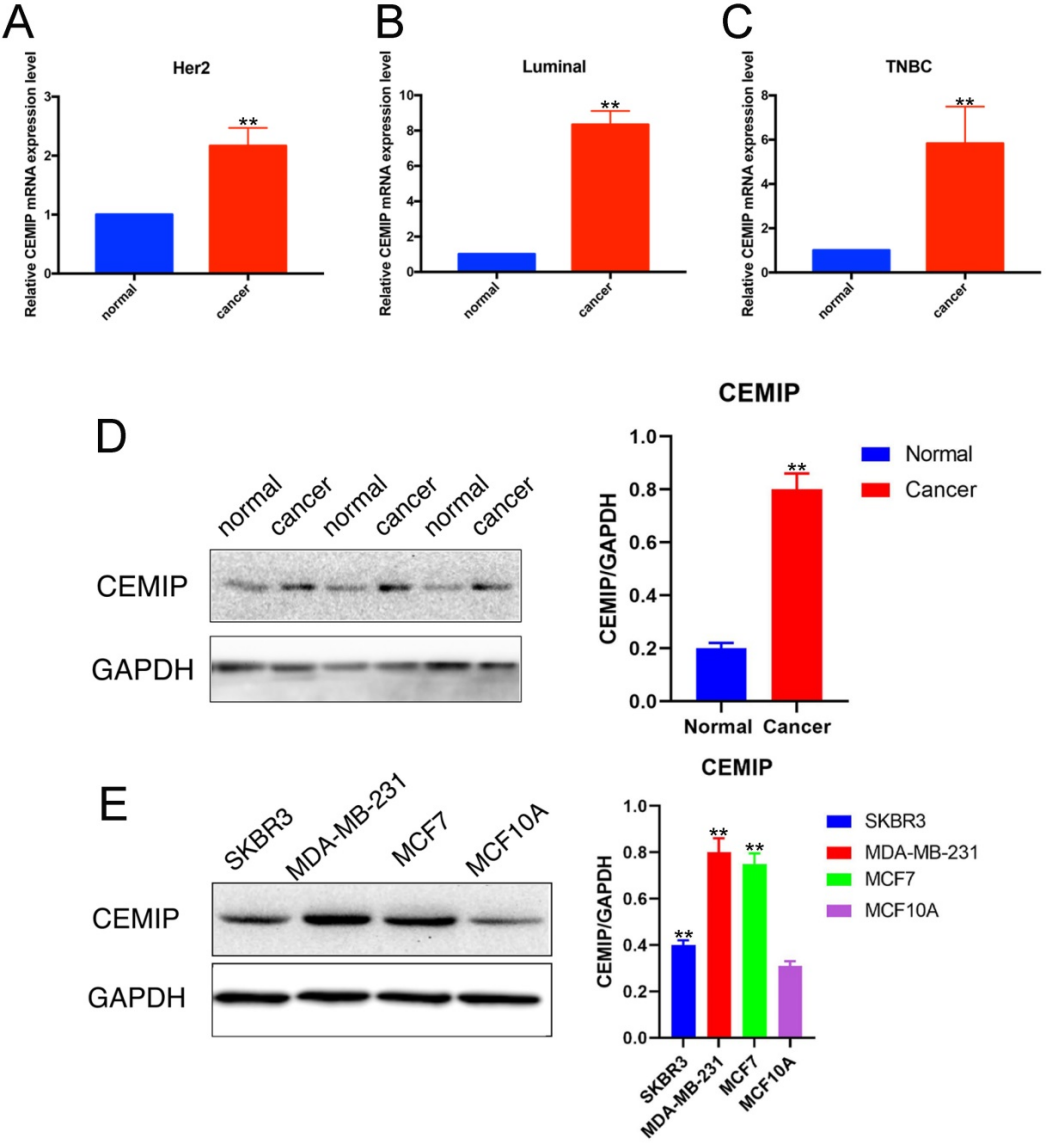
** Detection of CEMIP expression in BC samples and cell lines. A-C:** Detection of *CEMIP* mRNA expression in BC samples of different molecular subgroup. **D:** Detection of CEMIP expression in BC tissues and normal breast tissues specimens. **E:** Detection of CEMIP expression in different BC cell lines.

We detected the relative RNA expression of *CEMIP* in nine fresh paired BC samples and nontumor tissues via qRT-PCR. As shown in Figure 4A-4C, in three subtypes of BC,* CEMIP* expression was significantly higher in BC tissues than in nontumor tissues of BC different subtypes (*P* < 0.05). This result predicted that the expression of *CEMIP* mRNA was higher in BC tissues than in normal tissues regardless of different BC subtypes. And among different BC subtypes, there was little difference. In accordance with our PCR and western blotting analysis results for both cancer cell lines and clinical samples, *CEMIP* expression was found to be higher in cancer tissues than cancer-adjacent tissues (Figure 4D) and was also higher in the BC cell lines MDA-MB-231, SKBR3, and MCF7 than in the normal BC cell line MCF10A (Figure 4E). The basic clinicopathological characteristics of each cancer patients of Figure 4D were shown in Supplementary Table 3. About these four breast cell lines, MDA-MB-231 belonged to triple negative breast cancer cell lines. SKBR3 was HER2 positive breast cancer cell lines. MCF7 belonged to luminal breast cancer cell lines. MCF10A was normal BC cell line.

**Figure 5:**
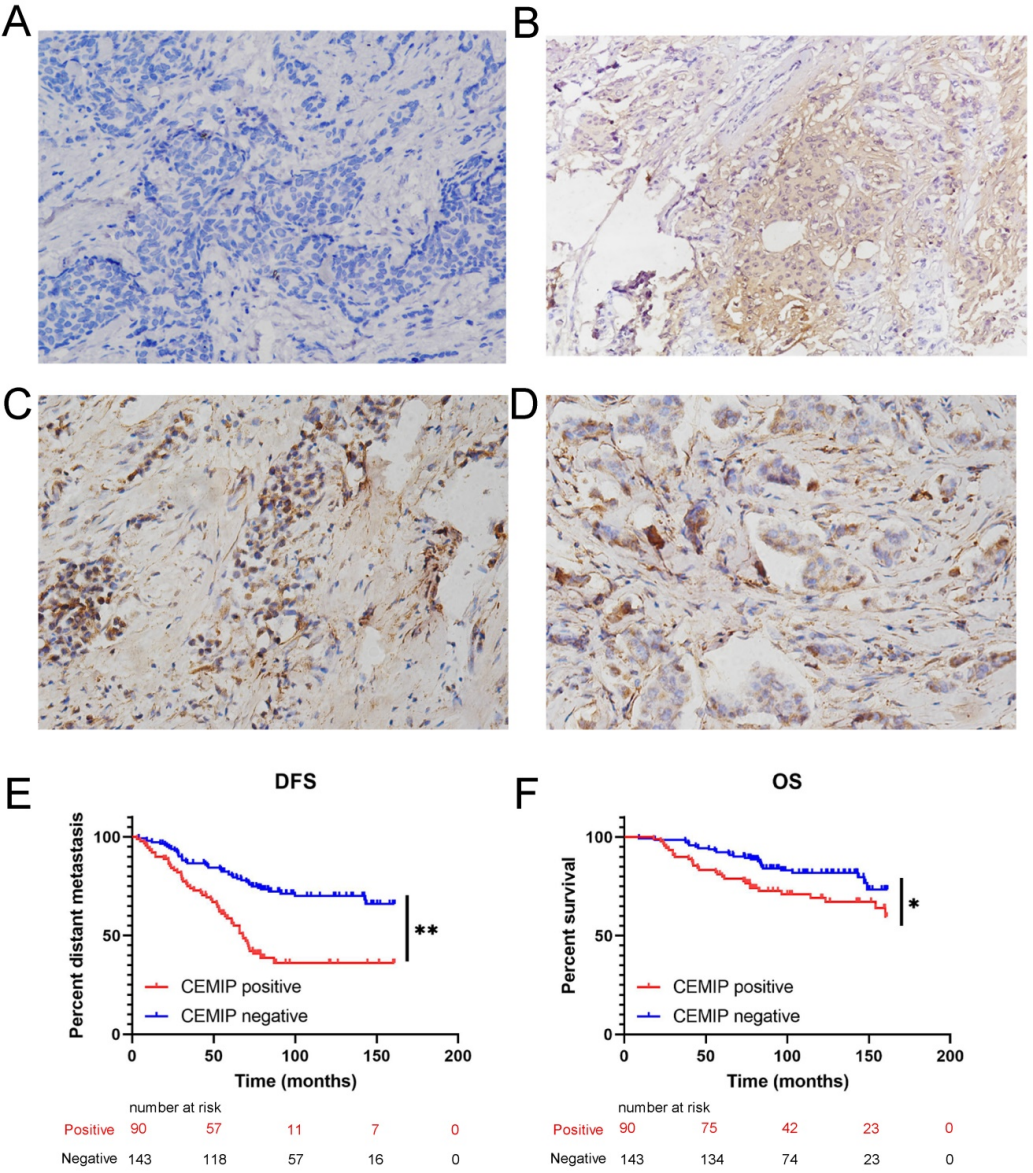
** Validation the prognostic effect of CEMIP expression in clinical BC specimens. A:** Representative negative CEMIP immunohistochemical results in BC specimens (×200 magnification). **B:** Representative positive CEMIP immunohistochemical results in luminal BC specimens (×200 magnification). **C:** Representative positive CEMIP immunohistochemical results in HER2+ BC specimens (×200 magnification). **D:** Representative positive CEMIP immunohistochemical results in triple negative BC specimens (×200 magnification). **E:** Validation the prognostic effect of CEMIP expression on the disease-free survival of BC patients. **F:** Validation the prognostic effect of CEMIP expression on the overall survival of BC patients.

Furthermore, a total of 233 female patients who underwent BC surgery were analyzed. The correlations between *CEMIP* expression and clinical characteristics are shown in Table 1. *CEMIP* expression was significantly associated with a higher ratio of distant metastasis (*P* < 0.001) and death (*P* = 0.014). A total of 90 out of the 233 patients (38.6%) were found to be CEMIP-positive via immunohistochemistry; typical pictures of *CEMIP*-negative and *CEMIP*-positive results in luminal, HER2+ and TNBC are shown in Figure 5A-5D. We subsequently compared the clinical and histological characteristics between positive (scores 1-3) and negative CEMIP expression (score 0) groups. However, no significant differences were identified in age, menopausal status, or other characteristics as shown in Table 1.

Furthermore, we evaluated the effect of CEMIP expression on the prognosis of 233 BC patients. We found that high CEMIP expression was related to a significantly shorter DFS (*P* < 0.001, Figure 5E) and OS (*P* = 0.016, Figure 5F).

### Genetic variations of *CEMIP* gene in BC

**Figure 6:**
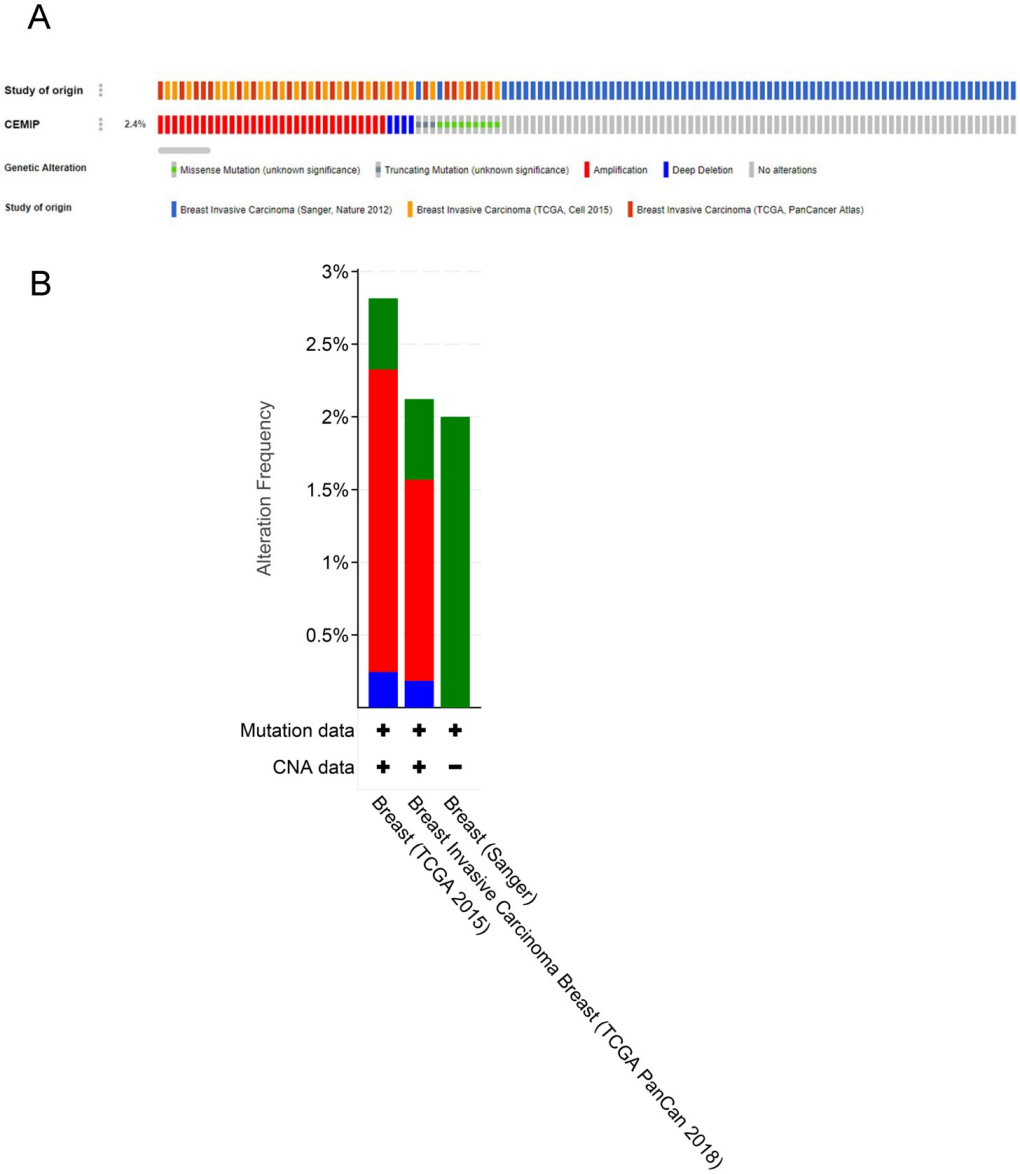
** Analyses of genetic variations of *CEMIP* by cBioPortal database. A:** OncoPrint visual summary of genetic variations of *CEMIP* in BC. **B:** Analyses of genetic variations of *CEMIP* in different BC studies.

Genetic variations of *CEMIP* in three studies (Sanger, Nature 2012; TCGA, Cell 2015; and TCGA, PanCancer Atlas) were analyzed using the cBioPortal database (Figure 6A). In these cases from TCGA, Cell 2015, the ratio of *CEMIP* mutation, amplification, and deep deletion was 2.8%, whereas in these cases from TCGA, PanCancer Atlas, the ratio of *CEMIP* mutation, amplification, and deep deletion was 2.1%. In these cases from Sanger, Nature 2012, the ratio of *CEMIP* mutations was 2% (Figure 6B).

### Effect DNA methylation on *CEMIP* mRNA expression

**Figure 7:**
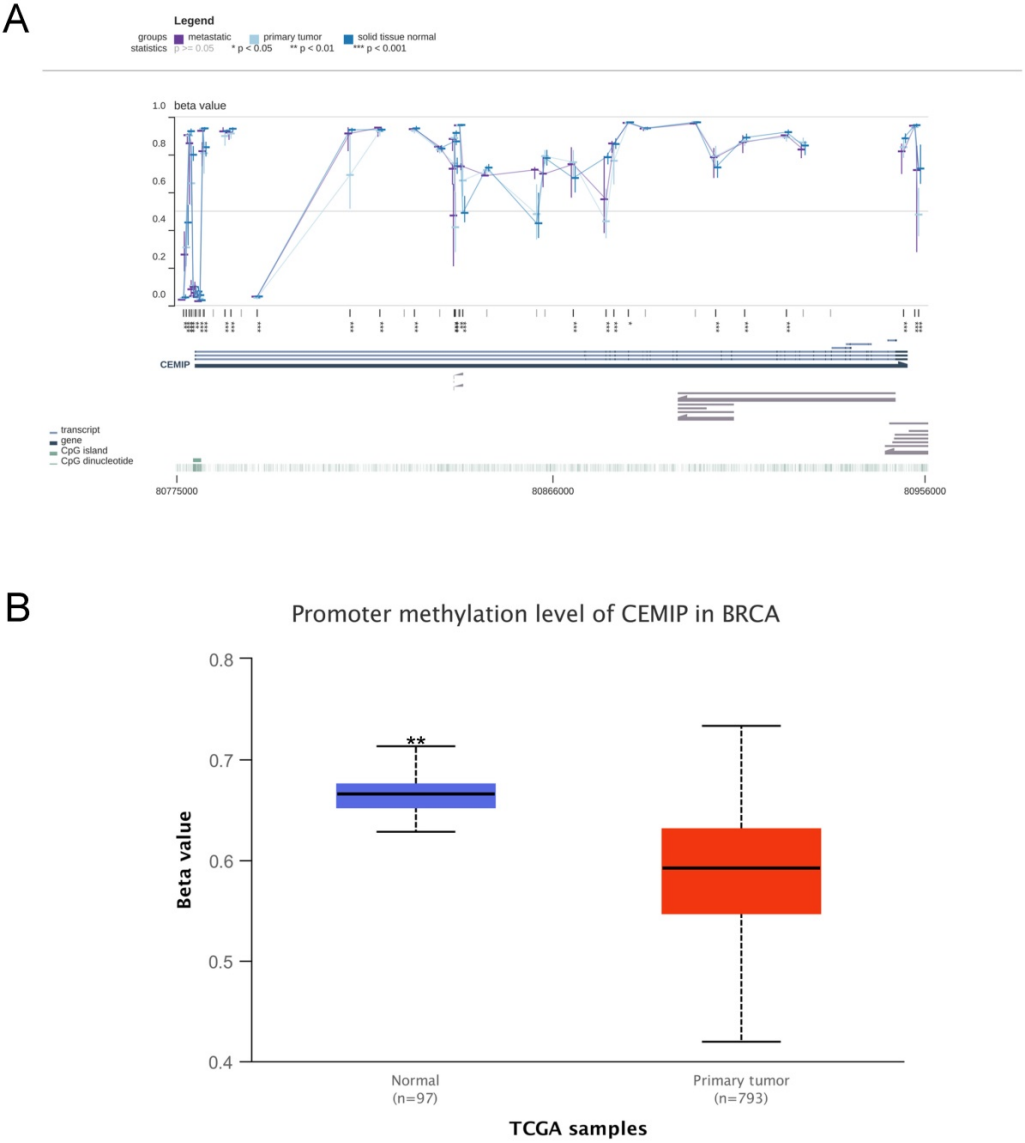
** Analyses of *CEMIP* promoter methylation in BC. A:** Analysis the level of *CEMIP* promoter methylation in primary BC tissues and normal breast tissues in MEXPRESS database. **B:** Analysis the level of *CEMIP* promoter methylation in primary BC tissues and normal breast tissues in UALCAN database.

In the MEXPRESS and UALCAN databases, the level of DNA methylation in *CEMIP* was higher in normal tissues than in cancer tissues (Figure 7A-7B). This result was consistent with the theory that DNA methylation is negatively related to mRNA expression 27. This result led to the prediction that *CEMIP* mRNA expression is regulated by DNA promoter methylation in BC.

### Regulation of immune-related molecules by *CEMIP* in BC

The immune-related molecules, including lymphocytes, immune inhibitors, immune stimulators, MHC molecules, chemokines, and chemokine receptors, which were regulated by *CEMIP* in BC, were further explored by using the TISIDB database.

Supplementary Figure 1A shows the relationship between* CEMIP* expression and tumor-infiltrating lymphocytes. The tumor-infiltrating lymphocytes most closely related with *CEMIP* expression in BC were CCL7, CCL14, CCL20, and CXCL14 (Supplementary Figure 1B). Supplementary Figure 1C shows the relationship between *CEMIP* expression and immune inhibitors. The immune inhibitors most closely related with *CEMIP* expression in BC were CD160, HAVCR2, PDCD1LG2, and TGFBR1 (Supplementary Figure 1D). Supplementary Figure 1E shows the relationship between *CEMIP* expression and immune stimulators. The immune stimulators most closely related with *CEMIP* expression in BC were CD276, TNFSF4, NT5E, and ULBP1 (Supplementary Figure 1F). Supplementary figure 1G shows the relationships between *CEMIP* expression and MHC molecules. The MHC molecules most closely related with *CEMIP* expression in BC were HLA-DMB, HLA-DOB, HLA-DOA1, and TAPBP (Supplementary Figure 1H).

Supplementary Figure 2A shows the relationship between *CEMIP* expression and chemokines. The chemokines most closely related with *CEMIP* expression in BC were CCL1, CCL19, CXCL18, and CXCL12 (Supplementary Figure 2B). Supplementary Figure 2C shows the relationships between *CEMIP* expression and chemokine receptors. The chemokine receptors most closely related with *CEMIP* expression in BC were CCR1, CCR4, CCR7, and CCR8 (Supplementary Figure 2D).

### Exploration of *CEMIP* molecular functions and regulation mechanism

In the Oncomine coexpression analysis derived from Ginestier's research, *CEMIP* expression was significantly correlated with SULF1 (r = 0.811) and PLAU (r = 0.830) 28 (Supplementary Figure 3A); Schmidt's research showed the same result for PLAU (r = 0.681) and SULF1 (r = 0.622) (Supplementary Figure 3B) 29. Lu's study shows that SULF1 has a poor survival outcome in ER-positive BC in the Chinese population 30, and PLAU is a key pathway protein in aggressive BC 31. Therefore, as a coexpression protein of SULF1 and PLAU, we speculate that *CEMIP* also plays an important role in TNBC. Using the bioinformatic databases, we selected several neighboring genes that were related to *CEMIP* from Coexpedia to explore the potential molecular mechanisms of the role of *CEMIP* in cancer and other diseases (Supplementary Figure 3C). The results show that PLAU is the most relevant protein with regard to *CEMIP*, which is consistent with our finding that *CEMIP* is a novel biomarker in BC.

Further, we explored the *CEMIP* molecular function and regulation pathways in BC. First, we explored these genes that interacted with *CEMIP* by the STRING database (Supplementary Figure 3D). These selected genes were then subjected to GO enrichment analysis (Supplementary Figure 3E). The GO analysis suggested that these proteins were mainly involved in the hyaluronan biosynthetic process, hyaluronan catabolic process, and other related processes. These findings may assist us to determine the exact regulatory mechanisms of *CEMIP* in BC.

## Discussion

CEMIP which has a PbH1 domain consisting of parallel β-helix repeats, is predicted to take part in polysaccharide hydrolysis process. This protein structure suggested that *CEMIP* is a kind of secreted factor that may play an important role in extracellular ligand binding and processing. Deregulated expression of *CEMIP* has been found in pancreatic tumors, prostate tumors, renal tumors, and breast tumors 32-35. However, the effect of *CEMIP* expression on the survival of BC patients, and related regulation mechanism remains unclear. In the study, *CEMIP* expression negatively influenced the survival outcomes of BC patients was found. The regulatory mechanism of *CEMIP* in BC was also explored.

Most of the aforementioned results strongly suggest that *CEMIP* serves as a negative factor for cancer prognosis. Specifically, the Oncomine analysis and TCGA data showed a higher level of *CEMIP* mRNA transcripts in BC tissues than in noncancerous tissues. Using clinical BC specimens, we found *CEMIP* expression was associated with a larger tumor size, distant metastasis, and even death. Additionally,* CEMIP* expression can also significantly shorten DFS and OS. Furthermore, we validated *CEMIP* mRNA levels in both BC cell lines and clinical samples. The results revealed that the *CEMIP* mRNA level was increased in BC tissues compared with normal breast tissues. Western blotting and database analysis showed that *CEMIP* was significantly highly expressed in carcinomas compared to nontumor tissues. *CEMIP* was overexpressed in MDA-MB-231, SKBR3, and MCF7 cells than MCF10A cells, indicating that *CEMIP* may be associated with metastasis and invasion of BC.

In addition, we found that DNA promoter methylation was involved in the transcription of *CEMIP*, although it was not a direct result. And CEMIP can regulate immune-related molecules to participate in BC progression. Additionally, Oncomine coexpression analysis showed expression of CEMIP as well as other BC oncogenes. Finally, using GO analysis, we found that *CEMIP* was significantly enriched in hyaluronan biosynthetic process and hyaluronan catabolic process and related processes. All these findings predicted that *CEMIP* is an adverse prognostic factor of BC and may participate in the regulation of BC progression.

This research also has some limitations. Firstly, this was a small sample study. Studies that include more BC patients are needed to explore the precise influence of *CEMIP* expression in BC. Secondly, we only explored the regulatory pathway of *CEMIP* based on bioinformatic tools and did not validate by *in vitro* cell experiments and *in vivo* animal experiments. Thus, future studies should focus on molecule experiments to elucidate the mechanisms by which *CEMIP* expression can affect the progression of BC.

Overall, this study found that *CEMIP* expression is higher in cancer tissues than in noncancerous tissues and that high *CEMIP* expression is associated with distant metastasis and death. And CEMIP can regulate immune-related molecules in BC. In conclusion, *CEMIP* was an adverse independent predictor of BC prognosis and may serve as a potential biomarker for BC patients.

## Supplementary Material

Supplementary figures and tables.

## Figures and Tables

**Table 1:** The correlations between CEMIP expression and clinicopathological characteristics

Variables	CEMIP positive (%)	CEMIP negative (%)	*P*-value
No. of patients	90 (38.6)	143 (61.4)	
**Age**			0.615
≤65	80 (88.9)	130 (90.9)	
>65	10 (11.1)	13 (9.1)	
**T grade**			0.110
1	24 (26.7)	51 (35.7)	
2	55 (61.1)	84 (58.7)	
3	11 (12.2)	8 (5.6)	
**N grade**			0.367
0	48 (53.3)	81 (56.6)	
1	19 (21.1)	38 (26.6)	
2	7 (7.8)	9 (6.3)	
3	16 (17.8)	15 (10.5)	
**Menopausal status**			0.380
Premenopausal	40 (44.4)	72 (50.3)	
Postmenopausal	50 (55.6)	71 (49.7)	
**ER status**			0.518
Positive	61 (67.8)	91 (63.6)	
Negative	29 (32.2)	52 (36.4)	
**PR status**			0.816
Positive	53 (58.9)	82 (57.3)	
Negative	37 (41.1)	61 (42.7)	
**HER2 status**			0.316
Positive	30 (33.3)	57 (39.9)	
Negative	60 (66.7)	86 (60.1)	
**Ki67 index**			0.676
≤20%	39 (43.3)	58 (64.4)	
>20%	51 (56.7)	85 (94.4)	
**Distant metastasis**			<0.001
Yes	52 (57.8)	42 (29.4)	
No	38 (42.2)	101 (70.6)	
**Death**			0.014
Yes	29 (32.2)	26 (18.2)	
No	61 (67.8)	117 (81.8)	

## Abbreviations

BC: breast cancer
CEMIP: cell migration-inducing and hyaluronan-binding protein
HA: hyaluronic acid
TNBC: triple-negative BC
OS: overall survival
PPS: post-progression survival
ATCC: American Type Culture Collection
TBS: Tris-buffered saline
DFS: disease-free survival
