# *CEMIP* as a potential biomarker and therapeutic target for breast cancer patients: Erratum

**DOI:** 10.7150/ijms.114765

**Published:** 2025-08-29

**Authors:** Jinqi Xue, Xudong Zhu, Xinbo Qiao, Yulun Wang, Jiawen Bu, Xiaoying Zhang, Qingtian Ma, Lu Liang, Lisha Sun, Caigang Liu

**Affiliations:** Department of Oncology, Shengjing Hospital of China Medical University, Shenyang, Liaoning Province, 110004, China.

The authors regretfully acknowledge that an incorrect image panel was inadvertently included in Figure 4D of the originally published article. To ensure the accuracy and integrity of the article, the authors have carefully reviewed the original data and replaced the erroneous image. All the authors have confirmed that the correction made in this erratum does not affect the original findings or conclusions of the study. We apologize for this error.

The revised Figure 4D and its corresponding caption are provided below.

## Figures and Tables

**Figure 4:**
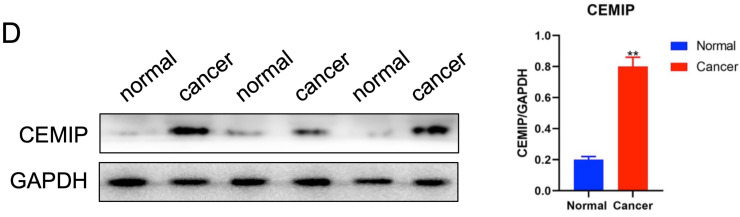
** Detection of CEMIP expression in BC samples and cell lines.** D: Detection of CEMIP expression in breast cancer tissues and normal breast tissue specimens. The WB bands shown are representative results from three of the twenty total specimens analyzed, and the statistic analysis image is based on the results of total 20 pairs of samples, as described in the Methods section.

