# Supplementary material for: CEMIP as a potential biomarker and therapeutic target for breast cancer patients
Source: Int J Med Sci. 2022 Feb 7;19(3):434–45. doi: 10.7150/ijms.58067 (PMC8964326; doi:10.7150/ijms.58067)
Supplement: Supplementary file 1 — Supplementary figures and tables. [file ijmsv19p0434s1.pdf]

Supp Fig 1

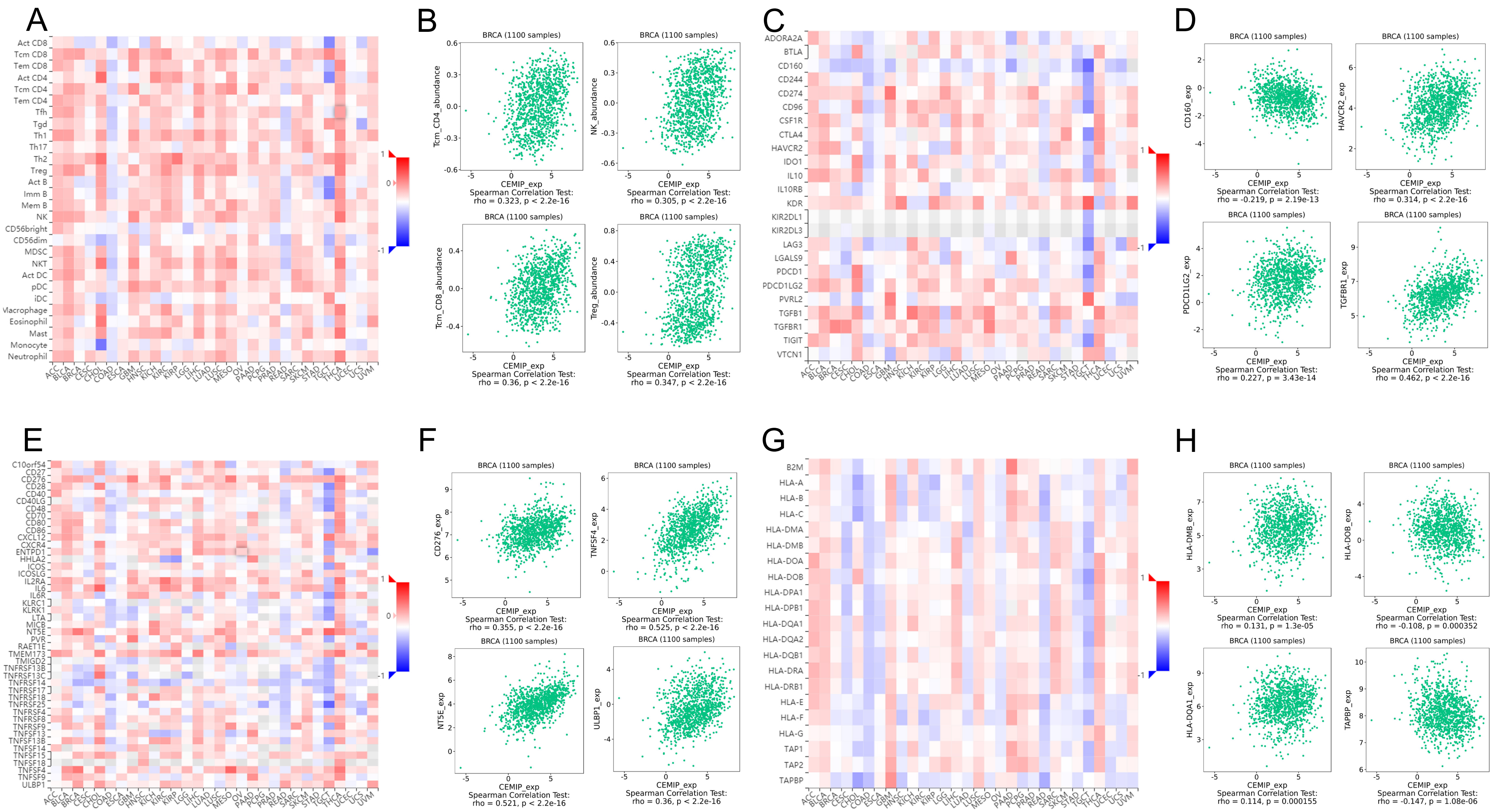

A
Act CD8
Tcm CD8
Tem CD8
Act CD4
Tcm CD4
Tem CD4
Tfh
Tgd
Th1
Th17
Th2
Treg
Act B
Imm B
Mem B
NK
CD56bright
CD56dim
MDSC
NKT
Act DC
pDC
iDC
Macrophage
Eosinophil
Mast
Monocyte
Neutrophil
ACC BLCA BRCA CESC CHOL COAD ESCA GBM HNSC KICH KIRC KIRP LGG LIHC LUAD LUSC MESO OV PAAD PCPG PRAD READ SARC SKCM STAD TGCT THCA UCEC UCS UVM
B
BRCA (1100 samples)
Tcm_CD4_abundance
CEMIP_exp
Spearman Correlation Test:
rho = 0.323, p < 2.2e-16
BRCA (1100 samples)
NK_abundance
CEMIP_exp
Spearman Correlation Test:
rho = 0.305, p < 2.2e-16
BRCA (1100 samples)
Tcm_CD8_abundance
CEMIP_exp
Spearman Correlation Test:
rho = 0.36, p < 2.2e-16
BRCA (1100 samples)
Treg_abundance
CEMIP_exp
Spearman Correlation Test:
rho = 0.347, p < 2.2e-16
C
ADORA2A
BTLA
CD160
CD244
CD274
CD96
CSF1R
CTLA4
HAVCR2
IDO1
IL10
IL10RB
KDR
KIR2DL1
KIR2DL3
LAG3
LGALS9
PDCD1
PDCD1LG2
PVRL2
TGFB1
TGFBR1
TIGIT
VTCN1
D
BRCA (1100 samples)
CD160_exp
CEMIP_exp
Spearman Correlation Test:
rho = -0.219, p = 2.19e-13
BRCA (1100 samples)
HAVCR2_exp
CEMIP_exp
Spearman Correlation Test:
rho = 0.314, p < 2.2e-16
BRCA (1100 samples)
PDCD1LG2_exp
CEMIP_exp
Spearman Correlation Test:
rho = 0.227, p = 3.43e-14
BRCA (1100 samples)
TGFBR1_exp
CEMIP_exp
Spearman Correlation Test:
rho = 0.462, p < 2.2e-16
E
C10orf54
CD27
CD276
CD28
CD40
CD40LG
CD48
CD70
CD80
CD86
CXCL12
CXCR4
ENTPD1
HHLA2
ICOS
ICOSLG
IL2RA
IL6
IL6R
KLRC1
KLRK1
LTA
MICB
NT5E
PVR
RAET1E
TMEM173
TMIGD2
TNFRSF13B
TNFRSF13C
TNFRSF14
TNFRSF17
TNFRSF18
TNFRSF25
TNFRSF4
TNFRSF8
TNFRSF9
TNFSF13
TNFSF13B
TNFSF14
TNFSF15
TNFSF18
TNFSF4
TNFSF9
ULBP1
F
BRCA (1100 samples)
CD276_exp
CEMIP_exp
Spearman Correlation Test:
rho = 0.355, p < 2.2e-16
BRCA (1100 samples)
TNFSF4_exp
CEMIP_exp
Spearman Correlation Test:
rho = 0.525, p < 2.2e-16
BRCA (1100 samples)
NT5E_exp
CEMIP_exp
Spearman Correlation Test:
rho = 0.521, p < 2.2e-16
BRCA (1100 samples)
ULBP1_exp
CEMIP_exp
Spearman Correlation Test:
rho = 0.36, p < 2.2e-16
G
B2M
HLA-A
HLA-B
HLA-C
HLA-DMA
HLA-DMB
HLA-DOA
HLA-DOB
HLA-DPA1
HLA-DPB1
HLA-DQA1
HLA-DQA2
HLA-DQB1
HLA-DRA
HLA-DRB1
HLA-E
HLA-F
HLA-G
TAP1
TAP2
TAPBP
H
BRCA (1100 samples)
HLA-DMB_exp
CEMIP_exp
Spearman Correlation Test:
rho = 0.131, p = 1.3e-05
BRCA (1100 samples)
HLA-DOB_exp
CEMIP_exp
Spearman Correlation Test:
rho = -0.108, p = 0.000352
BRCA (1100 samples)
HLA-DQA1_exp
CEMIP_exp
Spearman Correlation Test:
rho = 0.114, p = 0.000155
BRCA (1100 samples)
TAPBP_exp
CEMIP_exp
Spearman Correlation Test:
rho = -0.147, p = 1.08e-06

Supp Fig 2

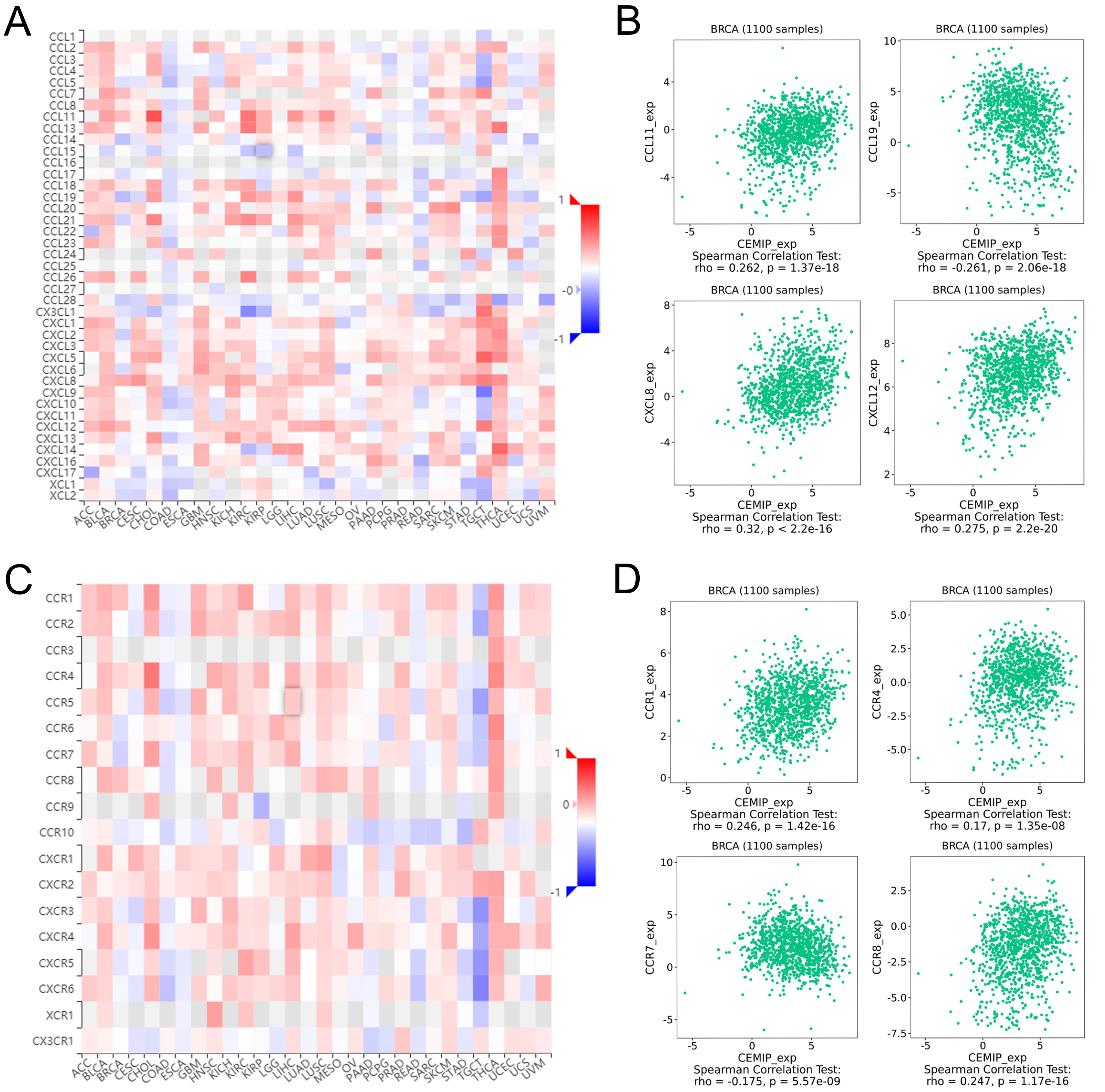
A
CCL1
CCL2
CCL3
CCL4
CCL5
CCL7
CCL8
CCL11
CCL13
CCL14
CCL15
CCL16
CCL17
CCL18
CCL19
CCL20
CCL21
CCL22
CCL23
CCL24
CCL25
CCL26
CCL27
CCL28
CX3CL1
CXCL1
CXCL2
CXCL3
CXCL5
CXCL6
CXCL8
CXCL9
CXCL10
CXCL11
CXCL12
CXCL13
CXCL14
CXCL16
CXCL17
XCL1
XCL2
ACC BLCA BRCA CESC CHOL COAD ESCA GBM HNSC KICH KIRC KIRP LGG LIHC LUAD LUSC MESO OV PAAD PCPG PRAD READ SARC SKCM STAD TGCT THCA UCEC UCS UVM
1
-0
-1
B
BRCA (1100 samples)
CCL11_exp
CEMIP_exp
Spearman Correlation Test:
rho = 0.262, p = 1.37e-18
BRCA (1100 samples)
CCL19_exp
CEMIP_exp
Spearman Correlation Test:
rho = -0.261, p = 2.06e-18
BRCA (1100 samples)
CXCL8_exp
CEMIP_exp
Spearman Correlation Test:
rho = 0.32, p < 2.2e-16
BRCA (1100 samples)
CXCL12_exp
CEMIP_exp
Spearman Correlation Test:
rho = 0.275, p = 2.2e-20
C
CCR1
CCR2
CCR3
CCR4
CCR5
CCR6
CCR7
CCR8
CCR9
CCR10
CXCR1
CXCR2
CXCR3
CXCR4
CXCR5
CXCR6
XCR1
CX3CR1
ACC BLCA BRCA CESC CHOL COAD ESCA GBM HNSC KICH KIRC KIRP LGG LIHC LUAD LUSC MESO OV PAAD PCPG PRAD READ SARC SKCM STAD TGCT THCA UCEC UCS UVM
1
0
-1
D
BRCA (1100 samples)
CCR1_exp
CEMIP_exp
Spearman Correlation Test:
rho = 0.246, p = 1.42e-16
BRCA (1100 samples)
CCR4_exp
CEMIP_exp
Spearman Correlation Test:
rho = 0.17, p = 1.35e-08
BRCA (1100 samples)
CCR7_exp
CEMIP_exp
Spearman Correlation Test:
rho = -0.175, p = 5.57e-09
BRCA (1100 samples)
CCR8_exp
CEMIP_exp
Spearman Correlation Test:
rho = 0.247, p = 1.17e-16

Supp Fig 3

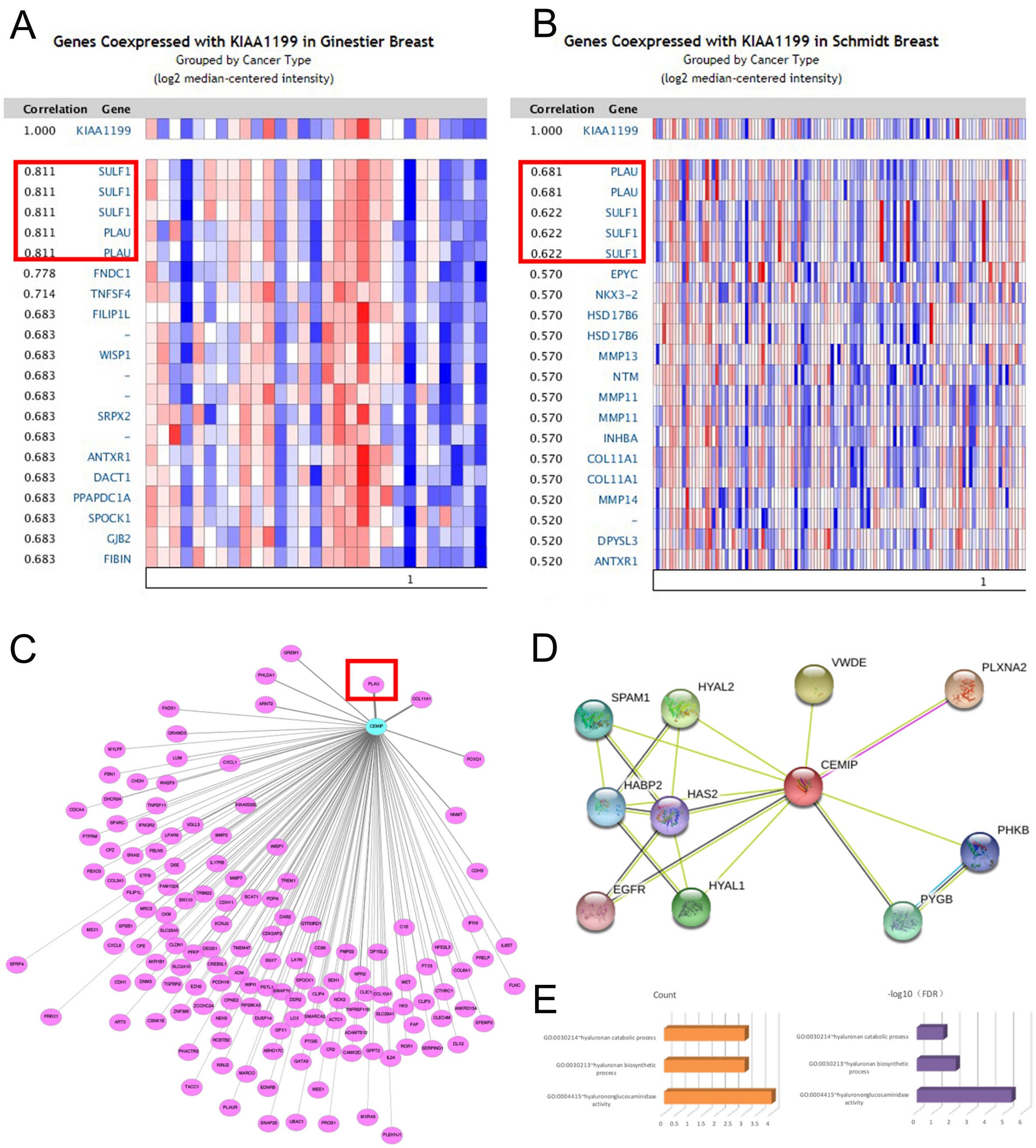
A
Genes Coexpressed with KIAA1199 in Ginestier Breast
Grouped by Cancer Type
(log2 median-centered intensity)
Correlation Gene
1.000 KIAA1199
0.811 SULF1
0.811 SULF1
0.811 SULF1
0.811 PLAU
0.811 PLAU
0.778 FNDC1
0.714 TNFSF4
0.683 FILIP1L
0.683 -
0.683 WISP1
0.683 -
0.683 -
0.683 SRPX2
0.683 -
0.683 ANTXR1
0.683 DACT1
0.683 PPAPDC1A
0.683 SPOCK1
0.683 GJB2
0.683 FIBIN
1
B
Genes Coexpressed with KIAA1199 in Schmidt Breast
Grouped by Cancer Type
(log2 median-centered intensity)
Correlation Gene
1.000 KIAA1199
0.681 PLAU
0.681 PLAU
0.622 SULF1
0.622 SULF1
0.622 SULF1
0.570 EPYC
0.570 NKX3-2
0.570 HSD17B6
0.570 HSD17B6
0.570 MMP13
0.570 NTM
0.570 MMP11
0.570 MMP11
0.570 INHBA
0.570 COL11A1
0.570 COL11A1
0.520 MMP14
0.520 -
0.520 DPYSL3
0.520 ANTXR1
1
C
D
VWDE
PLXNA2
HYAL2
SPAM1
CEMIP
HABP2
HAS2
PHKB
EGFR
HYAL1
PYGB
E
Count
-log10（FDR）

Supp table 1 Univariate and multivariate cox regression analyses of clinicopathological factors for DFS among these breast cancer patients

Variables	DFS			
	Univariate analysis		Multivariate analysis	
	HR (95%CI)	*P*-value	HR (95%CI)	*P*-value
Age	1.861(1.112-3.112)	0.018	2.200(1.290-3.753)	0.004
T grade			NS	
1		0.003		
2	0.763(0.490-1.187)	0.230		
3	2.267(1.193-4.307)	0.012		
N grade				
0		<0.001		<0.001
1	1.294(0.754-2.219)	0.349	1.542(0.893-2.664)	0.120
2	3.755(1.912-7.371)	<0.001	3.393(1.722-6.685)	<0.001
3	4.492(2.688-7.506)	<0.001	4.244(2.460-7.321)	<0.001
Menopausal status	1.414(0.938-2.133)	0.098	NA	
ER status	1.362(0.875-2.120)	0.172	NA	
PR status	1.326(0.884-1.988)	0.173	NA	
HER2 status	1.238(0.801-1.911)	0.337	NA	
Ki67 index	1.284(0.853-1.934)	0.231	NA	
CEMIP expression	2.751(1.824-4.151)	<0.001	3.062(1.986-4.721)	<0.001

NA: Non-analysis; NS: Non-significant

Supp table 2 Univariate and multivariate cox regression analyses of clinicopathological factors for OS among these breast cancer patients

Variables	OS			
	Univariate analysis		Multivariate analysis	
	HR (95%CI)	*P*-value	HR (95%CI)	*P*-value
Age	1.199(0.653-2.200)	0.558	NA	
T grade			NS	
1		0.036		
2	1.805(0.936-3.481)	0.078		
3	3.194(1.303-7.827)	0.011		
N grade				
0		<0.001		<0.001
1	1.970(0.979-3.965)	0.057	2.208(1.088-4.483)	0.028
2	5.439(2.232-13.225)	<0.001	4.750(1.879-12.010)	0.001
3	4.881(2.482-9.601)	<0.001	4.162(2.003-8.650)	<0.001
Menopausal status	1.301(0.763-2.218)	0.334	NA	
ER status	1.227(0.713-2.109)	0.460	NA	
PR status	2.110(1.235-3.608)	0.006	NS	
HER2 status	2.653(1.531-4.597)	0.001	NS	
Ki67 index	3.106(1.781-5.415)	<0.001	NS	
CEMIP expression	1.899(1.117-3.229)	0.018	1.823(1.055-3.149)	0.031

NA: Non-analysis; NS: Non-significant

Supp table 3 The basic clinicopathological characteristics of each cancer patients of Figure 4D

Characteristics	Patient 1	Patient 2	Patient 2
Age	51	49	56
T stage	2	2	2
N stage	1	1	1
subtype	Luminal type	Luminal type	Luminal type

Supp table 4 The relationships between CEMIP expression and the level of tumor-infiltration lymphocytes (TILs).

	CEMIP expression			*P*-value	R value
The level of TILs	Negative	Positive	Total		
Low	97	47	144	0.017	0.156
High	46	43	89		
Total	143	90	233		
